# Deep Learning-Based Concrete Surface Damage Monitoring Method Using Structured Lights and Depth Camera

**DOI:** 10.3390/s21082759

**Published:** 2021-04-14

**Authors:** Hyuntae Bang, Jiyoung Min, Haemin Jeon

**Affiliations:** 1 Department of Civil and Environmental Engineering, Hanbat National University, Dongseodae-ro 125, Daejeon 34158, Korea; htbang@hanbat.ac.kr; 2 Sustainable Infrastructure Research Center, Korea Institute of Civil Engineering and Building Technology, Goyangdae-ro 283, Goyang 10223, Korea; amote83@kict.re.kr

**Keywords:** damage detection, quantification, deep learning, structured light, depth camera

## Abstract

Due to the increase in aging structures and the decrease in construction workforce, there is an increasing interest in automating structural damage monitoring. Surface damage on concrete structures, such as cracks, delamination, and rebar exposure, is one of the important parameters that can be used to estimate the condition of the structure. In this paper, deep learning-based detection and quantification of structural damage using structured lights and a depth camera is proposed. The proposed monitoring system is composed of four lasers and a depth camera. The lasers are projected on the surface of the structures, and the camera captures images of the structures while measuring distance. By calculating an image homography, the captured images are calibrated when the structure and sensing system are not in parallel. The Faster RCNN (Region-based Convolutional Neural Network) with Inception Resnet v2 architecture is used to detect three types of surface damage: (i) cracks; (ii) delamination; and (iii) rebar exposure. The detected damage is quantified by calculating the positions of the projected laser beams with the measured distance. The experimental results show that structural damage was detected with an F1 score of 0.83 and a median value of the quantified relative error of less than 5%.

## 1. Introduction

Due to the increase in aging structures and a dearth of construction labor, various attempts have been made to replace the current inspector-centered exterior monitoring method. With the rapid development of computer vision and camera hardware technologies, vision-based structural health monitoring techniques have become a subject of active research. Vision-based structural damage monitoring methods can detect and quantify damage using consistent and reliable criteria through image processing techniques. In addition, when it is applied to a mobile platform such as a drone, it is possible to access various parts of large civil structures that are difficult for inspectors to access.

Jahanshahi et al. detected structural damage using image processing techniques such as noise removal, contour detection, and color transform [1]. Abdel-Qader et al. applied Fast Haar Transform (FHT), Fast Fourier Transform (FFT), a Sobel filter, and Canny contour detection to fifty different concrete bridge images to verify the performance of each method [2]. Lattanzi et al. improved the speed and accuracy of crack detection by using Canny contour detection and classifying the detected contours using the k-means clustering algorithm [3]. Nishikawa et al. developed a crack detection filter that relies on genetic programming and performed quantification by calculating changes in pixel intensity [4].

As deep learning-based object detection techniques develop, studies on damage detection and quantification using such techniques are actively being conducted. Cha et al. proposed a method of detecting cracks by using a convolutional neural network (CNN), a deep learning technique that extracts image features using a filter and classifies the image by simplifying [5]. In a follow-up study, a deep learning model based on Faster RCNN, which improves speed by integrating a network that generates candidate regions, is used to detect corrosion and peeling of steel materials, corrosion of bolts, and cracks [6]. Kim et al. proposed a surface crack monitoring method based on region-based convolutional neural network (RCNN) and quantified the cracks using markers [7]. Park et al. proposed a crack detection and quantification method using you only look once (YOLO) detector and a structured light [8]. Due to the installation or manufacturing error of the laser beams, the positions of the laser beams are pre-calculated, and the beams are calibrated using a laser distance sensor. Kim et al. proposed the use of Mask-RCNN to detect cracks and quantify them using the intrinsic parameters of the camera [9].

The aforementioned studies all have one or more of the following limitations: (i) the method is only capable of detecting damage on the structure; (ii) an external marker is necessary to quantify the detected damage; (iii) the accuracy of the quantification is only guaranteed when the sensing system and the structure are in parallel; and/or (iv) split-detected damages are not considered. Herein, to overcome these limitations, the rotation of the camera is estimated by using four laser beams and calculating the homography matrix, and the distorted images are corrected by applying the rotation angles of the *X* and *Y* axes. Multiple images of a single instance of damage are integrated using a stitching technique. The rest of the paper is organized as follows. In Section 2, the Faster RCNN model for identification of three types of damage is described. Damage quantification using a depth camera and lasers, including the sensor calibration method, is introduced in Section 3. To validate the performance of the proposed method, experimental tests were conducted, and the results are discussed in Section 4. Conclusions and further research are described in Section 5.

## 2. Deep Learning-Based Damage Detection

### 2.1. Recapitulation of Faster RCNN

Deep learning, one of the backbones of artificial intelligence, is undergoing remarkable progress, and is capable of outstanding performance in object detection in computer vision. Object detection is an automated technique that determines the presence of objects within predefined categories and separates them from the background. The positions of the object and their confidence levels are returned by the algorithm. Convolutional neural network (CNN) has played a pivotal role in the object detection problem that has been highlighted by deep learning. The CNN uses filters to perform convolution in each part of the image. Then, each feature of the image is extracted by filtering the points that showed a sudden change. The CNN is advantageous because it can be configured as a layer with multiple filters. Multiple types of features can be extracted using several different types of filters. In addition, the CNN is extremely helpful for object detection because it can correct errors while learning by combining the extracted features [10].

A CNN has several layers that are combined to form a single network. The layers of CNN are a convolutional layer, an activation layer, a pooling layer, a fully connected layer, and a softmax layer [11]. In the convolutional layer, a filter set with weights is defined through parameters. The parameters of the filter set attempt to prevent excessive redundancy detection and control the interval of filter application. In addition, the output data are padded so that they have the same size as the input data; this is done by filling the outside of the image with zeros to prevent loss of information. The activation layer controls the transfer of output values from the previous layer to the next layer. Sigmoid and rectified linear unit (ReLU) functions are applied to control whether the output value is transferred to the next layer. The sigmoid function is subject to the vanishing gradient problem, which causes the weight to disappear over a certain range. The ReLU function has greatly advanced the use of CNNs by solving the vanishing gradient problem by making the function linear in the range above zero [12]. The pooling layer uses the output value of the activation layer as a parameter to output the representative value within the defined filter range. The maximum value, average value, L2-norm, etc. are used as representative values. The computational load is reduced when the size of the data decreases as they go through the pooling layer. In addition, overfitting can be prevented because noise is eliminated in the process of extracting the representative values. The fully connected layer categorizes the features extracted by the convolutional and pooling layers. The artificial neural network is constructed by converting a two-dimensional array, the output of the convolutional layer, to a one-dimensional array. In the fully connected layer, the features extracted through previous layers are classified. The softmax layer calculates a probability for each category and outputs the result with the highest probability. Since the CNN, which is composed of various layers in combination, can extract image features through various filters, it has the advantage of being able to construct a general-purpose detection model that is relatively independent of the size or position of an object.

However, object detection using CNN has the disadvantage of long computational time. A lot of computational resources go into identifying the region of interest (RoI) containing the object because a sliding window algorithm is used to check all parts of the image at regular intervals. Therefore, to reduce the computational time, region with CNN features (RCNN) including selective search, an algorithm that proposes the RoI, was developed. Selective search divides regions according to features in the input image and examines the divided regions. Object detection is performed by combining similar regions by extracting and inspecting feature points using a CNN for the divided regions [13]. However, RCNN also takes a long time because the CNN-based calculation is performed on all initially divided RoIs. Fast RCNN was proposed to address this problem. The Fast RCNN saves computational resources by extracting features using the CNN and performing segmentation instead of segmenting the input image using the feature changes [14]. However, since the selective search algorithm is still used for region proposal, the method is still computational resource-intensive. To solve this issue, a Faster RCNN that uses a region proposal network (RPN) instead of the selective search algorithm was proposed. To extract the RoI from the image through CNN, a RPN was added for RoI extraction prior to object detection.

In the object detection model using CNN described above, the RoI detection process and the object classification process are performed separately. In contrast, with the case of you look only once (YOLO) or single shot multibox detector (SSD), RoI detection and object classification are simultaneously performed in one network. YOLO creates a grid by dividing the entire image by a certain ratio without going through the region proposal process and extracts an ROI for each grid. This has the advantage of reducing repetitive calculations and increasing search speed [15]. SSD uses a method similar to YOLO to extract bounding boxes with different scales with several layers and aggregates them to perform object detection. This can reduce computational time and improve accuracy [16]. In general, there is a tradeoff between detection speed and accuracy in object detection models [17]. In this paper, a detection model is constructed using Faster RCNN which detects the objects with higher accuracy.

### 2.2. Damage Identification Model Based on Faster RCNN

The Faster RCNN model was used to construct the surface damage detection model, which has the following three steps. The first step is a CNN that includes a convolutional layer and a max pooling layer to extract the features of the image. As the input image goes through the convolutional layer, the feature map is extracted. As it goes through the max pooling layer, the computational cost is reduced, and overfitting is prevented. The image that passed through the CNN is output as a feature map, and the output feature map is transferred to the RPN. In the second step, the RPN proposes the RoI containing the object. The RPN is a network that proposes a part that may have surface damage based on the feature map extracted through the CNN. Multiple anchor boxes each of a different size are used to detect the presence of damage. The predicted region of damage proposed by the RPN is transferred to the feature map created by the CNN. The final step is the classification, which determines the surface damage and the probability that the damage is present. The location of the damage is identified and extracted by combining the feature map obtained through the CNN in the previous step and the location information of the damage proposed through the RPN. Surface damage is finally detected by determining the existence and probability of damage using a classifier that targets the area where the extracted damage is likely to exist.

To increase efficiency and speed at the beginning of training, transfer learning, which uses a model trained on the COCO dataset, is used. The transfer learning is a method of setting the initial value of the model by using the weight of the previously trained model instead of a random weight when training starts. As a method of performing training while updating the weights based on the weights of the trained model, this method has the advantage that the training time can be shortened compared to using an arbitrary initial value [18]. Inception Resnet v2, a CNN structure composed of Inception modules, is used for feature extraction [19]. The convolutional layers constituting the Inception module each have different filters, which enables efficient learning by extracting and combining various features.

## 3. Damage Quantification Using Structured Lights and a Depth Camera

In this paper, the detected damage is quantified using lasers and a depth camera. Using the known pattern of the laser beams, the detected object can be transformed to world coordinates. In many cases, the lasers are not projected in parallel due to installation or manufacturing errors. To increase the accuracy of quantification, in this paper, the poses of the lasers are calibrated using a jig module and an extended Kalman filter. By using the calibrated positions of the laser beams with the distance obtained from the depth camera, a homography matrix can be estimated. The captured images are rotated with the calculated homography matrix when the camera and the surface of the structure are not vertical. From the undistorted image of the lasers and damage, the size of structural damage can be estimated by considering the calibrated positions of the lasers.

### 3.1. Calibration of the Structured Light

**Figure 1:**
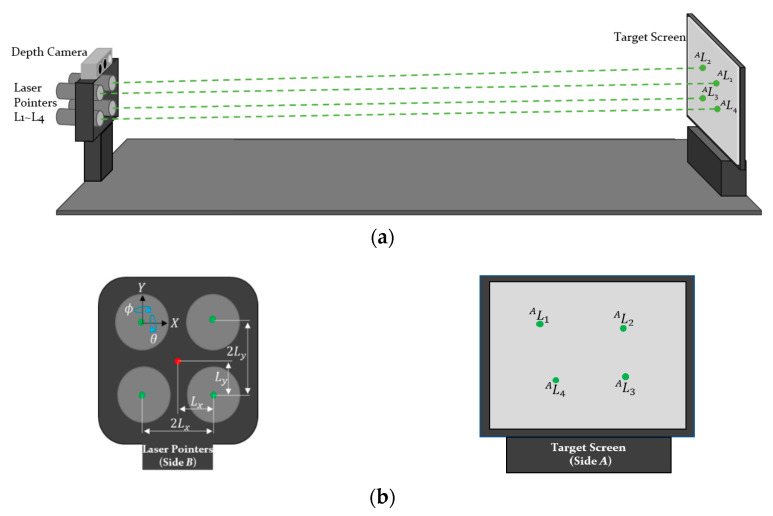
Design of the jig for laser calibration: (**a**) overall design of the jig module; and (**b**) magnified plot of each side in the jig module.

The specially designed jig module previously proposed by Park et al. is used to calculate the pose of lasers [8]. As shown in Figure 1, the laser beams are projected on the screen at a known distance *H*, and the camera captures an image of the screen. By considering the inclination angles of the lasers about the *X* and *Y* axes, the position of the projected laser beams on the screen can be calculated as follows:

(1) $${}^{A}L_{1}={}^{A}T_{B}\cdot {}^{B}T_{B’}\cdot {\left[L_{x}\;L_{y}\;0\;1\right]}^{T}$$

where *^A^L*_1_ is the projected laser beam on side *A* and *^A^T_B_* is the homogeneous transformation matrix which gives the relationship between coordinate frames of the screen (Σ*A*) and lasers (Σ*B*). As the jig geometry is given a priori, *^A^T_B_* is known. *^B^T_B’_* is the transformation consisting of inclination angles (see Equation (2)), and *L_x_* and *L_y_* are the *x* and *y* offsets of the installed lasers.

(2) $${}^{B}T_{B^{\prime }}=R_{x}(\theta_{L1})\cdot R_{y}\left(\phi_{L1}\right)$$

In Equation (2), *R_x_*(*θ*) indicates a rotation matrix along the *X*-axis with rotation angle *θ* and *θ_L1_* and *ϕ_L1_* are inclination angles of the laser, *L*_1_. Since the laser beams are projected on a 2D surface of the structure, the *z* components of *L*_1_ should be zero. Using the constraints, the kinematics of the initial pose of the lasers can be derived as in Equation (3) by combining Equations (1) and (2). Referring to Equation (1), the projected laser point, *^A^L*_1_, can be written as follows:

(3) $${}^{A}L_{1}=\left[\begin{array}{c} L_{x}c_{\phi_{L1}}-(s_{\phi_{L1}}(H+L_{y}s_{\theta_{L1}}-L_{x}c_{\theta_{L1}}s_{\phi_{L1}}))/(c_{\phi_{L1}}c_{\theta_{L1}})\\ L_{y}c_{\theta_{L1}}+s_{\theta_{L1}}(H+L_{y}s_{\theta_{L1}}-L_{x}c_{\theta_{L1}}s_{\phi_{L1}})/c_{\theta_{L2}}+L_{x}s_{\phi_{L1}}s_{\theta_{L1}}\\ 0\\ 1 \end{array}\right]$$

where *s_θ_* and *c_θ_* denote sin*θ* and cos*θ*, respectively. In a similar way, the kinematic equation including the positions of the four projected laser beams on Σ*A*, *^A^L_i_* (*i* = 1, 2, 3, and 4), can be derived as follows:

(4) $$\begin{array}{l} M={[}^{A}L_{1x}{\;}^{A}L_{1y}{\;}^{A}L_{2x}{\;}^{A}L_{2y}{\;}^{A}L_{3x}{\;}^{A}L_{3y}{\;}^{A}L_{4x}{\;}^{A}L_{4y}]\\ \;=\left[\begin{array}{c} \begin{array}{c} L_{x}c_{\phi_{L1}}-(s_{\phi_{L1}}(H+L_{y}s_{\theta_{L1}}-L_{x}c_{\theta_{L1}}s_{\phi_{L1}}))/(c_{\phi_{L1}}c_{\theta_{L1}})\\ L_{y}c_{\theta_{L1}}+s_{\theta_{L1}}(H+L_{y}s_{\theta_{L1}}-L_{x}c_{\theta_{L1}}s_{\phi_{L1}})/c_{\theta_{L2}}+L_{x}s_{\phi_{L1}}s_{\theta_{L1}}\\ L_{x}c_{\phi_{L2}}+s_{\phi_{L2}}(L_{y}s_{\theta_{L2}}-H+L_{x}c_{\theta_{L2}}s_{\phi_{L2}})/(c_{\phi_{L2}}c_{\theta_{L2}})\\ -L_{y}c_{\theta_{L2}}-s_{\theta_{L2}}(L_{y}s_{\theta_{L2}}-H+L_{x}c_{\theta_{L2}}s_{\phi_{L2}})/c_{\theta_{L2}}+L_{x}s_{\phi_{L2}}s_{\theta_{L2}} \end{array}\\ -L_{x}c_{\phi_{L3}}-s_{\phi_{L3}}(H-L_{y}s_{\theta_{L3}}+L_{x}c_{\theta_{L3}}s_{\phi_{L3}})/(c_{\phi_{L3}}c_{\theta_{L3}})\\ s_{\theta_{L3}}(H-L_{y}s_{\theta_{L3}}+L_{x}c_{\theta_{L3}}s_{\phi_{L3}})/c_{\theta_{L3}}-L_{y}c_{\theta_{L3}}-\;L_{x}s_{\phi_{L3}}s_{\theta_{L3}}\\ \begin{array}{c} -L_{x}c_{\phi_{L4}}-s_{\phi_{L4}}(H+L_{y}s_{\theta_{L4}}+L_{x}c_{\theta_{L4}}s_{\phi_{L4}})/(c_{\phi_{L4}}c_{\theta_{L4}})\\ L_{y}c_{\theta_{L4}}+s_{\theta_{L4}}(H+L_{y}s_{\theta_{L4}}+L_{x}c_{\theta_{L4}}s_{\phi_{L4}})/c_{\theta_{L4}}-L_{x}s_{\phi_{L4}}s_{\theta_{L4}} \end{array} \end{array}\right] \end{array}$$

Using the extended Kalman filter (EKF) method, the initial inclination angles of the lasers, *P_L_* = [*θ_Li_*, *Φ_Li_*], where *i* = 1, 2, 3, and 4, can be estimated considering the measurement noise which occurs when detecting the center of the laser beams. By using the Newton–Raphson method [20], the inclination angles of the lasers *P_L_* = [*θ_Li_*, *Φ_Li_*], where *i* = 1, 2, 3, and 4, can be estimated iteratively as follows:

(5) $${\hat{P}}_{L}(k+1)={\hat{P}}_{L}(k)+J_{P}{}^{†}(m(k)-\hat{m}(k))$$

where *J_P_* is the Jacobian of the kinematic equation, *M*, *J_P_*^†^ is the pseudo-inverse of the Jacobian, and *m(k)* and $\hat{m}$*(k)* are measured and estimated observations by *M*, respectively. In the case that there exist uncertainties in the measurement, the EKF scheme can be applied to estimate the *P_L_*. In this paper, inclination angles are estimated by using the EKF considering the measurement noise which occurs when detecting the center of the laser beams.

### 3.2. Damage Quantification Via Structured Lights and A Depth Camera

**Figure 2:**
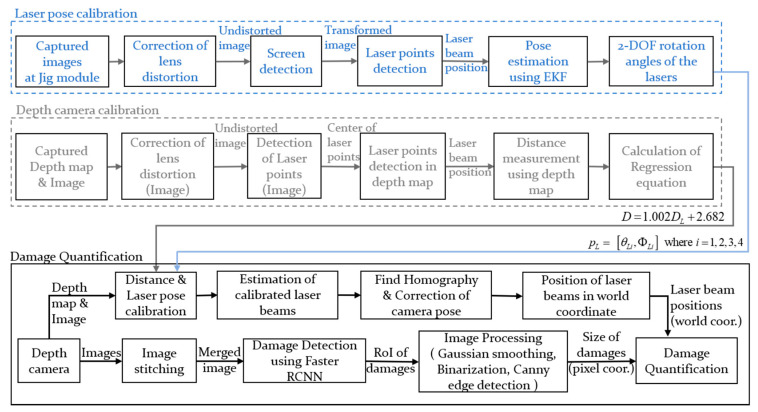
Schematic diagram of structural damage quantification.

The schematic diagram of damage quantification using structured lights and a depth camera is shown in Figure 2. After calibrating the initial inclination angles of the lasers, the homography matrix can be estimated from the calculated positions of the laser beams. By rotating the captured images considering the extrinsic parameters, the distorted images are corrected when the camera and the structure are not in parallel. In this paper, the calibrated images are merged when a single instance of damage is captured in multiple images. The positions of the laser beams are calculated using various image processing techniques such as Gaussian smoothing, image binarization, and canny detection. Gaussian smoothing, which reduces the high-frequency components on the image, is applied to increase the accuracy of contour detection. 

**Figure 3:**
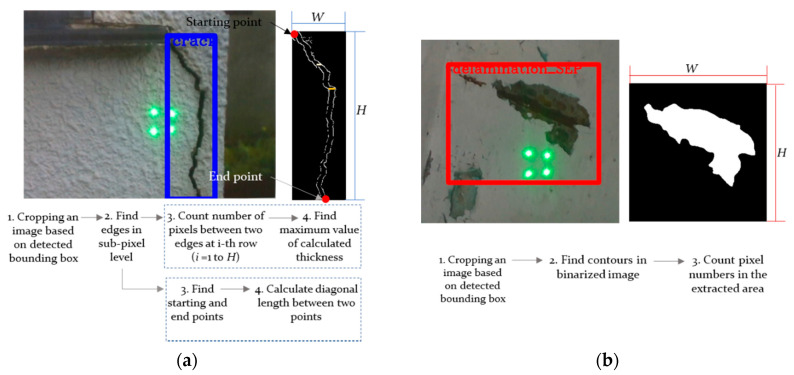
Image processing for quantification of: (**a**) cracks; and (**b**) delamination and rebar exposure.

The image processing for damage quantification is shown in Figure 3. As shown in the figure, the image is cropped based on the location of the bounding box and then image binarization with an adaptive thresholding method is performed. Afterwards, the contour of the damages is extracted. In the case of a crack, the coordinates of the starting point and the end point are found from the extracted edges; the diagonal length, which is considered as length in this paper, is calculated afterwards. The number of pixels between two edges is counted on every row in the longitudinal direction of the crack, and the largest value is set as the crack thickness. In the case of delamination or rebar exposure, the contour of the detected damage is extracted and the area is calculated. The length, width, or area of structural damage is translated into world coordinates based on the positions of the projected laser beams. In the process of calibrating the position of the laser beams based on the previously calculated initial inclination angles of the lasers, the distance between the lasers and the structure is required. The distance obtained from the depth camera is used after the correction.

## 4. Experimental Test

### 4.1. Experimental Setup

**Figure 4:**
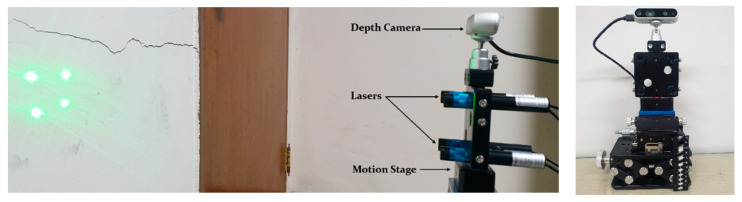
Experimental setup using a depth camera and lasers.

**Figure 5:**
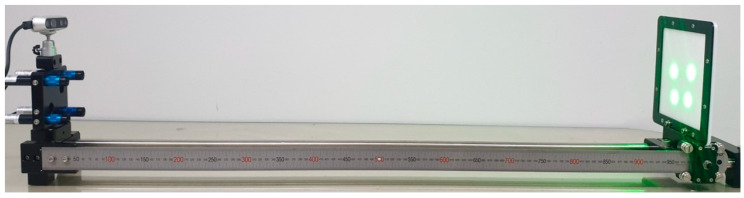
Laser pose calibration with a specially jig module.

To verify the proposed method for detecting and quantifying surface damage on concrete structures, experiments with structured lights and a depth camera were conducted. As shown in Figure 4, four lasers were projected on the concrete structures, and a depth camera on the same side of lasers captured the image of the structures while measuring the distance. The experiments used an LDD532-10-5 laser (Laserlab, Ltd.) and a Realsense D435i depth camera (Intel). The initial pose of the lasers was calibrated by using a specially designed jig module, as shown in Figure 5 [8]. The lasers were projected on the screen at the known distance, and the rotation angles of the lasers were calculated from the captured images using the extended Kalman filter. The estimated angles about the *X* and *Y* axes of lasers were [−0.47, 0.12], [−0.07, 1.04], [0.39, 0.86], and [0.73, 0.14], respectively. All units are in degrees.

**Figure 6:**
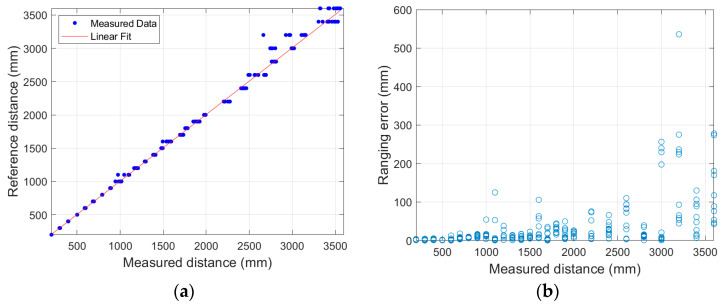
Distance measurements acquired through depth camera: (**a**) reference and measured distances with linear fit; and (**b**) ranging error caused by systematic bias.

**Figure 7:**
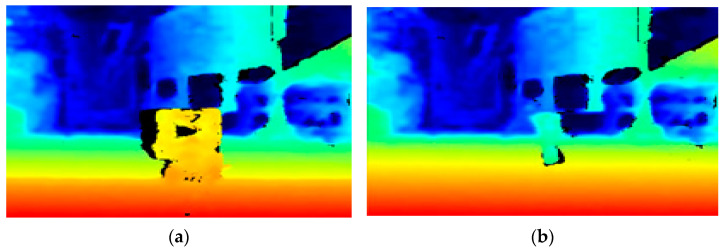
Depth maps at distances of: (**a**) 1000 mm; and (**b**) 3000 mm.

The Realsense D435i is a depth camera that calculates distance based on the difference between captured images through two lenses; the measured distance was used after analyzing the correcting the measurement information from each distance sensor [21]. To analyze the characteristics of the depth sensor, distances were measured through 10 repetitions at a constant distance. The measurement distance was calculated at intervals of 100 mm up to 200–2000 mm and at intervals of 200 mm up to 2000–3600 mm. The error according to the distance showed a tendency to increase rapidly above 3000 mm, as shown in Figure 6. When comparing the difference according to the distance of the depth map, which outputs the distance information acquired by the camera, the depth map at 1000 mm can clearly identify the target screen, as shown in Figure 7a. However, as shown in Figure 7b, in the depth map at 3000 mm, it is difficult to clearly identify the target as the boundary between the target screen and the background becomes blurred. Therefore, an experiment was conducted with the aim of quantification at distances of less than 3000 mm.

The regression equation for correcting the measured distance, which is based on the measured distances between the camera and the target screen, is shown in Equation (6). *D* is the corrected distance using the regression equation and *D_L_* is the measured distance. The R^2^ value of the regression equation is 0.9997 and the unit is in mm.

*D* = 1.002*D_L_* + 2.682 (6)

To construct a surface damage detection model for concrete structures, a dataset was constructed by collecting 1000 crack images, 1410 delamination images, and 292 exposed rebar images. Eighty percent of the dataset was used for learning to build the detection model, and the remaining images were randomly selected and used for verification of the detection model. 

The shorter is the distance between the camera and the structure, and the higher is the resolution of the captured image that can be obtained within the same area. Therefore, the image stitching technique was applied to quantify the structural damage with a high precision. In the stitching technique, the scale-invariant feature transform (SIFT) algorithm is used to detect feature points, and the k-nearest neighbors for each detected feature are found using a k-dimensional tree [22]. The feature points are matched by using random sample consensus (RANSAC) with finding the geometrical consistency. 

In total, 25 concrete surface damage images, including six crack images, eight delamination images, seven rebar exposure images, and four images with more than two types of damages, were used to verify the quantification performance. To evaluate the performance of distortion correction according to the angle when the camera and the structure are not parallel, the inclination angle of each image was set to 0, 5, and 10 degrees. The artificial inclines were applied using a rotational motion stage. In the case of a crack, quantification was performed by measuring the maximum thickness and the length; for delamination and rebar exposure, quantification was performed by measuring the damage area. The quantification results of cracks were evaluated against the ground truth measured using SC-10 and SC-20 crack scales (Sincon Co., Bucheon, Korea), which measures the thickness and length of each crack. The sizes of the damaged area were compared with the quantification results using a square marker [7]. 

### 4.2. Experimental Results

**Figure 8:**
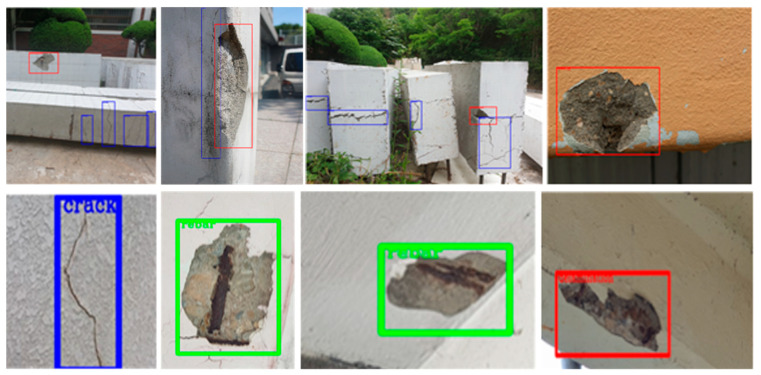
Damage detection using the Faster RCNN.

Using the Faster RCNN object detection model, a surface damage detection model was constructed by performing training using a training dataset consisting of 800 crack images, 1128 delamination images, and 233 rebar exposure images. The performance of the constructed surface damage detection model was verified using the remaining 20% of the dataset. The detection results using the deep learning algorithm are shown in Table 1 and Figure 8. As shown in the table, and the accuracy and F1 score of structural damage detection were 0.76 and 0.83, respectively.

**Figure 9:**
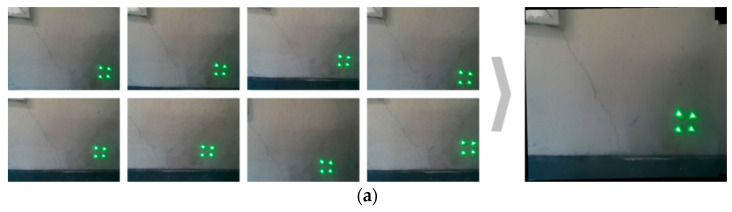

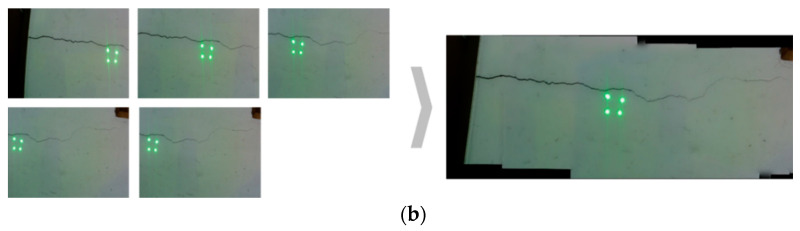
Image integration using the stitching algorithm. The captured images including (**a**) shear and (**b**) settlement cracks, and the merged image after performing homography calculation and image stitching.

To verify the performance of the quantification including the image stitching technique by considering the calibrated rotation angles of the camera, experimental tests with shear and settlement cracks were conducted. The split images were taken for each crack and homography calculation and image integration were performed, as shown in Figure 9. As shown in Table 2, the advantage of the laser pose calculation that calibrates the captured images is clearly seen.

To verify the performance of the proposed quantification system, an experiment was performed using 25 images of concrete surface damage, consisting of six crack images, eight delamination images, seven rebar exposure images, and four images with a combination of more than two types of damage. The damage was detected with Faster RCNN and Inception Resnet v2. Of the 32 instances of concrete surface damage in 25 images, 26 were detected. By type of damages, 10 cracks, 9 delaminations, and 7 rebar exposures were detected, which corresponds to a detection rate of 81.25% 

**Figure 10:**
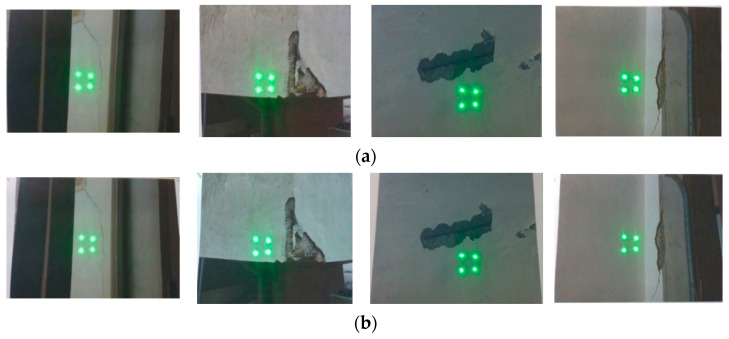

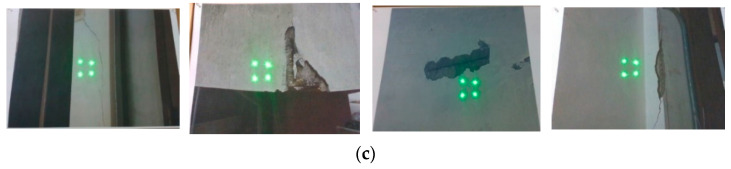
The captured images with the slope of: (**a**) 0 degrees; (**b**) 5 degrees; and (**c**) 10 degrees.

**Figure 11:**
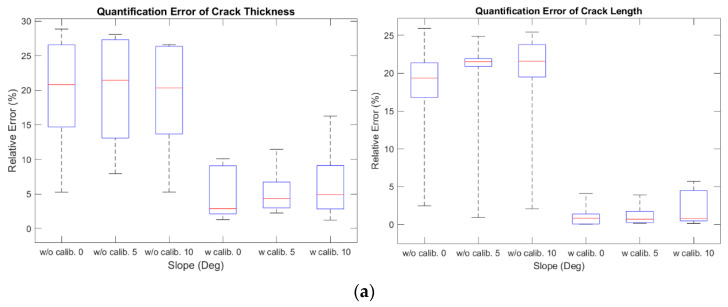

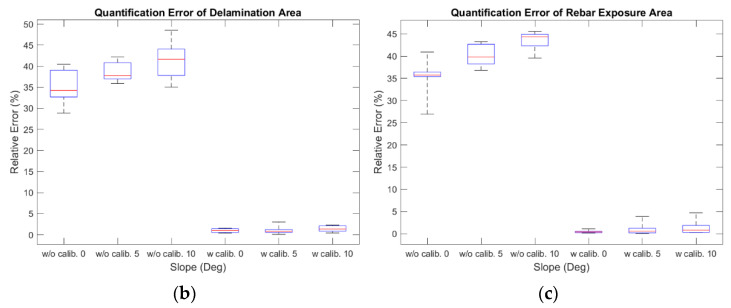
Boxplot of quantification results of structural damage by type. Relative error of: (**a**) thickness and length of cracks; (**b**) area of delamination; and (**c**) area of rebar exposure.

The experimental tests using three types of damages (cracks, delamination, and rebar exposure) were conducted based on the images obtained by varying the angle between the depth camera and the structure (0, 5, and 10 degrees) (see Figure 10). The relative error of the different types of damages and angles are shown in Table 3 and Figure 11. The median of the relative error for thickness and length of the crack were less than 4.87% and 0.83%, respectively; for areas of delamination, it was less than 1.35%; and for the area of rebar exposure, it was less than 0.79%. In comparison to the other types of damage, the relative error of crack thickness tends to be large. This is because the width of a crack is much smaller than the other types of damage, so even an error of the same absolute size is relatively large.

### 4.3. Discussions on the Experiments

Two kinds of experimental tests were conducted to verify the performance of the proposed method. In the first experimental test, the split images taken for each crack were integrated by image processing techniques such as SIFT and RANSAC. The detected cracks after applying image stitching technique were quantified by projecting four laser beams. The projected laser beams were calibrated by measuring the distance between the camera and the structure, and the previously estimated inclination angles of the lasers. The quantification results show that the thickness and length of integrated cracks are quantified with improved accuracy after the laser pose calibration. In the other experimental test, three types of damages, namely cracks, delamination, and rebar exposure, were used to verify the performance of detection and quantification techniques. The results show that the damages are detected with the rate of 81.25% and quantified with the median relative error of less than 5% under various conditions of the slope angles.

As shown in the experimental results, the proposed method detects and quantifies structural damages with a high level of accuracy by using the depth camera and lasers with calibrated inclination angles in comparison with previous studies [5,6,7,8,9]. By using the image stitching techniques, concrete surface damages with various sizes and shapes are reliably quantified with high precision. In addition, the sensing system for damage detection and quantification has been simplified compared to conventional methods using external markers or a distance sensor [7,8].

## 5. Conclusions

This paper describes a deep learning-based structural damage detection and quantification method using structured lights and a depth camera. Faster RCNN with Inception v2 is used to detect three types of structural damage: cracks, delamination, and rebar exposure. The detected damages are quantified using structured light and a depth camera. The projected laser beams are captured by the depth camera along with distance. Since the accuracy of quantification is directly related to the position of the projected laser beams, the initial inclination angles of lasers are calculated using a specially designed jig module. In addition, the homography is calculated and the images are calibrated when the structure and the sensing system are not in parallel. Quantification was performed based on the calibrated images and the positions of the laser beams considering the initial inclination angles, in which thickness and length were calculated in the case of cracks and area was calculated in the cases of delamination and rebar exposure. The experimental test results validate the proposed method, which was able to detect and quantify structural damage with an F1 score of 0.83 and a median relative error of less than 5%. In the future, the proposed method will be applied to various sizes and shapes of damages such as incipient damages including plastic shrinkage or settling cracks with a sensor module of the improved specifications. In addition, the filtering algorithm for reducing misclassification error in damage detection will be developed with an improved detection model.

## Figures and Tables

**Table 1:** Performance evaluation of the concrete damage detection model.

Accuracy	Average Precision	Average Recall	F1 Score
0.764815	0.93339	0.744322	0.828203

**Table 2:** Quantification results using integrated crack images.

Type of Cracks (Reference Value)	Thickness (T)				Length (L)			
	w/o Calibration		w Calibration		w/o Calibration		w Calibration	
	Meas (mm)	Error (%)	Meas (mm)	Error (%)	Meas (mm)	Error (%)	Meas (mm)	Error (%)
Shear crack (T: 1.6, W: 323.0)	1.58	1.25	1.61	0.63	316.57	1.99	322.59	0.13
Settlement crack (T: 3.5, W: 864.0)	2.16	38.29	3.55	1.43	618.84	28.38	884.04	2.32

**Table 3:** Relative error of quantification results according to slope (0, 5, and 10 degrees).

		Slope (Deg.)	Number of Samples	Min	Max	Median	First Quartile	Third Quartile
Crack thickness	Without calibration	**0**	10	5.25	28.84	20.82	14.68	26.56
		**5**	10	7.93	28.06	21.42	13.07	27.31
		**10**	10	5.26	26.60	20.33	13.68	26.33
	With calibration	**0**	10	1.27	10.09	2.85	2.11	9.07
		**5**	10	2.24	11.46	4.35	2.99	6.73
		**10**	10	1.22	16.25	4.87	2.83	9.11
Crack length	Without calibration	**0**	10	2.47	25.92	19.34	16.79	21.38
		**5**	10	0.93	24.88	21.50	20.90	21.92
		**10**	10	2.09	25.44	21.59	19.49	23.79
	With calibration	**0**	10	0.03	4.09	0.83	0.07	1.39
		**5**	10	0.16	3.89	0.71	0.24	1.76
		**10**	10	0.15	5.69	0.78	0.49	4.49
Delamination area	Without calibration	**0**	9	28.86	40.46	34.28	32.68	39.08
		**5**	9	35.89	42.22	37.77	36.96	40.85
		**10**	9	35.04	48.55	41.68	37.81	44.06
	With calibration	**0**	9	0.38	1.59	1.01	0.53	1.41
		**5**	9	0.09	3.02	0.77	0.56	1.24
		**10**	9	0.39	2.28	1.35	0.84	2.11
Rebar exposure area	Without calibration	**0**	7	26.93	40.91	35.73	35.40	36.40
		**5**	7	36.83	43.25	39.78	38.25	42.69
		**10**	7	39.59	45.54	44.31	42.34	44.90
	With calibration	**0**	7	0.15	1.09	0.40	0.23	0.53
		**5**	7	0.05	3.87	0.55	0.19	1.24
		**10**	7	0.28	4.73	0.79	0.28	1.88

## Data Availability

Data available on request due to restrictions eg privacy or ethical.
